# Improving the thermostability of alginate lyase FlAlyA with high expression by computer-aided rational design for industrial preparation of alginate oligosaccharides

**DOI:** 10.3389/fbioe.2022.1011273

**Published:** 2022-09-07

**Authors:** Xiu Zhang, Wei Li, Lixia Pan, Liyan Yang, Hongliang Li, Feng Ji, Yunkai Zhang, Hongzhen Tang, Dengfeng Yang

**Affiliations:** ^1^ College of Life Science and Technology, Guangxi University, Nanning, Guangxi, China; ^2^ Guangxi Key Laboratory of Marine Natural Products and Combinatorial Biosynthesis Chemistry, Guangxi Beibu Gulf Marine Research Center, National Engineering Research Center of Non-food Biorefinery, State Key Laboratory of Non-Food Biomass and Enzyme Technology, Guangxi Academy of Sciences, Nanning, Guangxi, China; ^3^ Viticulture and Wine Research Institute, Guangxi Academy of Agricultural Sciences, Nanning, Guangxi, China; ^4^ Institute of Medicine and Health Research, Guangxi Academy of Sciences, Nanning, Guangxi, China; ^5^ School of Pharmacy, Guangxi University of Chinese Medicine, Nanning, China

**Keywords:** alginate lyase, high-level expression, thermostability, rational design, molecular dynamics simulation, alginate oligosaccharide

## Abstract

FlAlyA, a PL7 alginate lyase with industrial potential, is widely applied in the preparation the alginate oligosaccharide because of its high activity of degradation the alginate. However, heat inactivation still limits the industrial application of FlAlyA. To further enhance its thermostability, a group of mutants were designed, according to evaluating the B-factor value and free energy change via computer-aided calculation. 25 single-point mutants and one double-points mutant were carried out by site-directed mutagenesis. The optimal two single-point mutants H176D and H71K showed 1.20 and 0.3°C increases in the values of *T*_m_, while 7.58 and 1.73 min increases in the values of half-life (*t*_1/2_) at 50°C, respectively, compared with that of the wild-type enzyme. Interestingly, H71K exhibits the comprehensive improvement than WT, including expression level, thermal stability and specific activity. In addition, the mechanism of these two mutants is speculated by multiple sequence alignment, structural basis and molecular dynamics simulation, which is likely to be involved in the formation of new hydrogen bonds and decrease the SASA of the mutants. These results indicate that B-factor is an efficient approach to improves the thermostability of alginate lyase composed of β-sheet unit. Furthermore, the highest yield of the mutant reached about 650 mg/L, which was nearly 36 times that of previous studies. The high expression, excellent activity and good thermal stability make FlAlyA a potential candidate for the industrial production of alginate oligosaccharides.

## Introduction

Marine algae as a non-food biomass has the characteristics of wide distribution, high oil content, strong environmental adaptability, short growth cycle, high yield, and no occupation of arable land. Alginate is the main polysaccharide of the brown algae cell wall, its content is approximately 30%–60% dry weight (Roesijadi et al., 2010). However, the widespread application of high molecular weight (Mw) alginates is greatly limited in view of the severe drawback of poor water solubility and low bioavailability (Lee and Mooney, 2012).

With the understanding and exploration of marine resources, various methods are available for the degradation of alginate into alginate oligosaccharides (AOS) with a variety of significant physiological characteristics such as antioxidant (Jiang et al., 2021), antitumor (Chen et al., 2017), anticancer (Fang et al., 2017), anti-inflammatory (Saigusa et al., 2015), immune regulation (Wang et al., 2021). The unsaturated monosaccharides would be converted into 4-deoxy-L-erythro-5-hexoseulose uronate (DEH), which can be used as the substrate to produce biofuel (Kawai and Hashimoto, 2022). In addition, the use of algae to produce biofuel has broad development prospects, and algae biofuel is likely to become one of the most important renewable energy sources in the future (Lee and Mooney, 2012).

Alginate lyases (Alys), one of polysaccharide lyases (PLs), could cleave 1,4-glycosidic bond between C4 and C5 through β-elimination reaction to depolymerize alginate into alginate oligosaccharides with different degrees of polymerization (Wong et al., 2000). Due to its high efficiency, substrate specificity and mild reaction conditions, alginate lyase has attracted more and more attention for its application in industrial production, especially in the production of AOS (Cheng et al., 2020).

Alginate lyases with stable performance are very valuable in the enzymatic production of alginate oligosaccharide in industrial production and have significant application prospects. Thermostable enzymes exhibit higher kinetic and operational stability during the process of alginate lyases catalysis and can continuously react at elevated temperature, resulting in reduced substrate viscosity, which is of great significance for the production of alginate oligosaccharides (Bloom et al., 2006; Suresh et al., 2021). This not only facilitates the efficient recycling of biocatalysts, speeds up the reaction rate and reduces the risk of microorganism contamination, but also reduces the time and energy required for cooling (Xu et al., 2020). In addition, in the production process of oligosaccharides, sufficient supply of alginate lyase can ensure high production efficiency, which requires high-yield expression of alginate lyase (Li et al., 2018).

Up to now, although many alginate lyases have been discovered, relatively few of them can meet industrial needs. Currently, the discovery of new thermostable alginate lyases from extreme environments (Inoue et al., 2016a), enzyme immobilization strategies (Li et al., 2019), and molecular modification of existing poorly thermostable enzymes through protein engineering have become the main approaches to obtain thermostable alginate lyases (Cao et al., 2022). Recently, some enzyme immobilization strategies have been developed for improvement of enzyme activities and protein stabilities (Wang et al., 2018; Liu et al., 2019; Zhang et al., 2021, 2022). However, the preparation of immobilized enzymes often requires complicated steps, and the scope of application of each immobilization method is different. Notably, the rational design in protein engineering is an important means to obtain mutants with desired properties based on the sequences and structures of enzymes, which has been successfully applied to improve the thermostability of enzymes (Wu et al., 2021).

However, to our knowledge, there are still few studies utilizing rational design to improve alginate lyase efficiency (Cao et al., 2022). Disulfide bonds can increase the structural rigidity and thermal stability of enzymes by reducing the entropy of protein expansion process, the thermostability of alginate lyases cAlyM and PyAly was improved by introducing disulfide bonds (Inoue et al., 2016a; Yang et al., 2019). However, the disulfide bond reduces the soluble expression of the protein to a certain extent, which is not conducive to the mass production of alginate lyase. Alginate lyases Aly7B and AlgH truncated the non-catalytic domain to improve thermostability (Hu et al., 2019; Yan et al., 2019). After truncating the non-catalytic domains of alginate lyase, the enzyme has a more compact three-dimensional structure that is more resistant to thermal denaturation conditions. However, the non-catalytic domain often plays a role in helping maintain enzyme activity, so the function of the non-catalytic domain must be thoroughly characterized before constructing truncations.

The alginate lyase FlAlyA secreted by *Flavobacterium* sp. UMI-01 has high enzymatic activity and good thermal stability (Inoue et al., 2014). Nippon Gene (Tokyo, Japan) marketed it as “HULK” in 2015, and 5 mg is priced at $238.43. In this work, targeted mutagenesis based on HotSpot Wizard 3.0 and dezyme web servers further improved the thermostability of the alginate lyase FlAlyA. And the highest yield of alginate lyase FlAlyA reached 650 mg/L culture. To better understand the mechanism of enhanced thermal stability, multiple sequence alignment, structural basis and molecular dynamics (MD) simulations were performed. Collectively, the strategy of improved thermostability based on rational design herein could be used as basis for further protein engineering, ultimately providing the excellent alginate lyase for industrial applications of alginate oligosaccharides.

### Marerials and methods

#### Construction of high-level expression plasmids pET-22b (+)-flAlyA

The alginate lyase gene *flAlyA*, from *Flavobacterium* sp. UMI-01 (GenBank accession number: BAP05660) was synthesized from Wuhan Gene Create Biological Engineering Co., Ltd. The synthesized DNA fragment harbored the restriction sites *Nde* I and *Xho* I was ligated into expression vector pET-22b (+) (Merck Millipore), named pET-22b (+)-*flAlyA*. The recombinant plasmid was then transformed into *E. coli* DH5α cells.

### Screening of the predicted flexible residues based on computer-aid design

The mutation sites were selected using Hotspot Wizard 3.0 (https://loschmidt.chemi.muni.cz/hotspotwizard/) based on B-factor analysis screening for non-conserved amino acid residues with high B-factor. And dezyme web server (https://soft.dezyme.com/) was used to screen flexible residues by calculating the ∆∆G value.

### Construction of mutants by site-directed mutagenesis

Single-point mutations of FlAlyA were constructed by site-directed mutagenesis using pET-22b (+)-*flAlyA* as the template. The double-point mutant Var (H71K + H176D) of FlAlyA was constructed by mutating H176 to D176 using pET-22b (+)-*flAlyA* ^H71K^ as template. All primers used for mutagenesis are listed in Supplementary Table S1. The PCR cycling program was set as follows: 1) 95°C, 15 min; 2) 95°C, 30 s; 60°C, 30 s; and 72°C, 7 min (35 cycles); and 3) 72°C, 10 min. After digesting the PCR product were incubated with restriction enzyme *Dpn*I to eliminate the methylated parental template, the purified PCR products were transformed into *E. coli* DH5α cells and the positive mutants were confirmed by the results of DNA sequencing (Sangon Biotech, China).

### Protein expression and purification of WT and its mutants

Recombinant wild-type FlAlyA and its mutants were overexpressed in BL21-CodonPlus (DE3)-RIL cells. The cells were first inoculated in Luria−Bertani (LB) medium containing 1 mM Ampicillin at 37°C and 220 rpm in a shaker incubator. Then, the overnight culture was transferred into fresh LB medium and cultivated at 37°C, 220 rpm. When the OD_600_ value reached 0.6–0.8, isopropyl-β-D-thiogalactopyranoside (IPTG) at a final concentration of 0.1 mM was added to induce the expression of the recombinant enzyme at 25°C for 24 h. Subsequently, the recombinant cells were harvested by centrifugation (7,200 rpm, 4°C, 10 min), and the pellet was resuspended in the lysis buffer (20 mM Tris-HCl, 300 mM NaCl, 20 mM imidazole, 10% glycerin, pH 7.4). Then, ultrasound treatments were performed (600 W, 10 min, work 2 s and stand 2 s) to release the recombinant enzymes. The supernatant was loaded onto a Ni-NTA column, equilibrated with buffer (20 mM imidazole, 25 mM Tris, pH 7.4, 500 mM NaCl) and eluted with elution buffer (500 mM imidazole, 25 mM Tris, 500 mM NaCl, pH 7.4). The protein concentration was determined using the NanoDrop 2000c Spectrophotometer (Thermo Fisher Scientific).

### Enzyme activity assay of WT and its mutants

Enzymatic activity was determined by measuring the increase in absorbance at 235 nm due to the formation of unsaturated double bonds at the nonreducing end of the sugar. The activity assays were performed in a buffer containing 0.5% (w/v) sodium alginate, 10 mM sodium phosphate (NaPi) buffer (pH 8.0), 100 mM NaCl, 0.1 mg/ml BSA, and 1 ug/ml FlAlyA at 30°C for 10 min. One unit of enzyme activity was defined as the amounts of enzyme that increase the absorbance at 235 nm by 0.01 per min.

### Enzyme thermostability determination of WT and its mutants

Only mutants without significant decrease of enzyme activity in comparison to the WT were further screened. To assess the thermostability of these mutants, their melting point temperature (*T*_m_) were characterized. Thermal shift assays were used to measure the *T*_m_. All of the proteins at 1 mg/ml in 10 mM NaPi buffer (pH 8.0) were incubated in 20 μL with 80×SYPRO Orange protein stain (Invitrogen, America). 7500 Fast Real-Time PCR System was used to conduct the experiment and fluorescence was detected over a temperature range of 25–95°C.

For the optimum temperature (*T*_*opt*_) determination, the enzyme activities were measured at various temperatures (from 30 to 70°C, with a temperature interval of 5°C) using the assay described in the Enzyme Activity Assay section. The highest enzyme activity of WT was set to 100%, and the activities of other temperatures and mutants were calculated as its relative (%) values.

The measurement method of the half-lives *t*_1/2_ value of thermal inactivation was as follows: the residual enzyme activity of each sample was measured at 50°C for different times, and the corresponding time was the half-life of the enzyme when the residual enzyme activity decreased to 50% of the untreated enzyme activity. The data were processed with Origin software to obtain *t*_1/2_ value. The enzymatic activity of the unincubated enzyme was set to 100%, and activities at the other times were calculated as relative (%) values to it.

The apparent kinetic parameters of WT and mutants were determined at different concentrations of sodium alginate (0.1%–4.0%) in 10 mM NaPi buffer (pH 8.0) with the addition of 0.1 mg/ml enzyme. The experimental data were fitted to the Michaeli−Menten kinetics model to obtain kinetic parameters using Origin. All assays were performed at least in triplicate.

### Circular dichroism analysis of WT and its mutants

Circular Dichroism (CD) spectra were measured with a Chirascan CD spectrometer (Applied PhotoPhysics) to determine the secondary structures of the proteins. The concentration of the purified and desalted enzyme solution was 0.4 mg/ml. The temperature was 25°C, the wavelength range was 190–260 nm with 1 nm intervals, and all measurements were performed in quartz cuvettes with a cell path length of 1 nm. The data were corrected by removing the control of buffer (10 mM NaPi buffer, pH 8.0).

### Molecular dynamics simulations of WT and its mutants

The conformation of FlAlyA was obtained from the Protein Data Bank (PDB) (https://www.rcsb.org/), and the PDB ID is 5Y33. The three-dimensional structures of the mutants were conducted using PyMol software based on crystal structure above. MD simulations were carried out to further evaluate the structural flexibility of the wild-type and the mutants. All MD simulations were performed with OpenMM (Peter and Vijay, 2010), a toolkit for molecular simulation, using the AMBER force field (Song et al., 2019). And the root-mean-square fluctuation (RMSF) and solvent accessible surface area (SASA) values were calculated.

## Results and discussion

### Screening of the potential residues for mutation via computer-aided design

**FIGURE 1:**
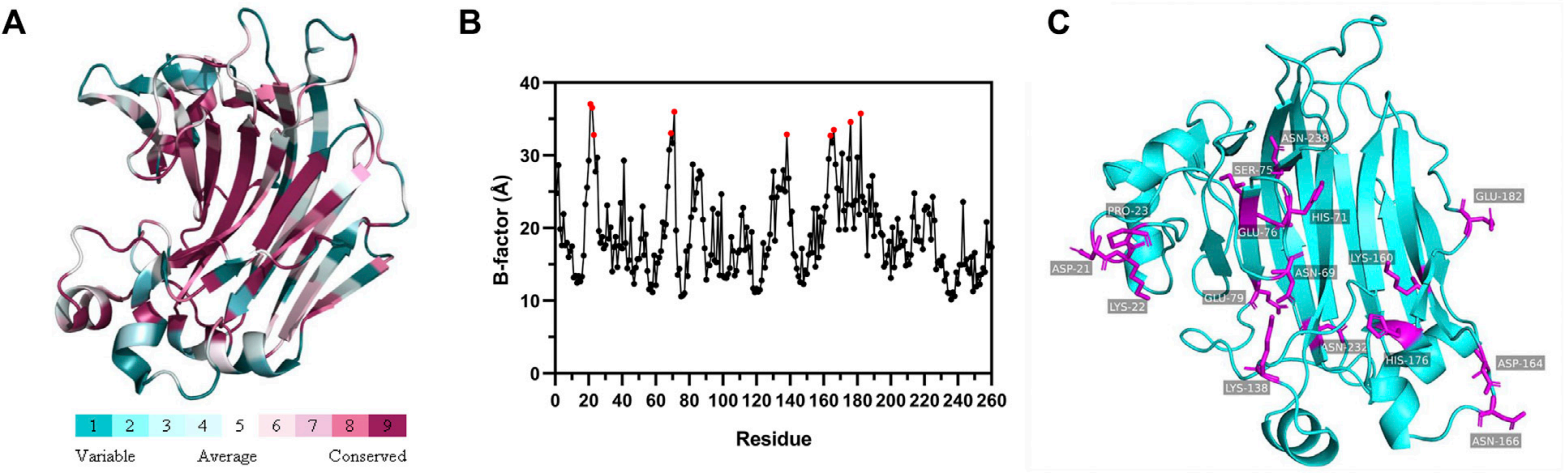
Flexible residues are determined based on the structure. **(A)** The evolutionary conservation is determined based on the structure and multiple sequence alignment. The amino acid residues are colored in gradient representing the conservation grades from the most variable (turquoise) to the most conserved (maroon) obtained with ConSurf program. **(B)** Identification of the flexible residue according to the B-factor values of the WT FlAlyA determined from the crystal structure (PDB: 5y33). The top ten sites with B-factor value were selected as mutation sites and marked with red dots. **(C)** Spatial location of all mutation sites in WT FlAlyA. Mutation amino acid residues are shown as stick models and marked in magenta.

To obtain mutants of FlAlyA with enhanced thermostability, we firstly rationally designed amino acid changes based on the analysis results of B-factor (≥32.73 Å) and ∆∆G (≤−1.10 kcal/mol) (Table 1). B-factor is an important parameter for evaluating protein stability. The larger the B-Factor value of the amino acid constituting the protein, the smaller the effect of the amino acid on stabilizing the protein (Sun et al., 2019). The Consurf Server (https://consurf.tau.ac.il/) enables analysis of protein conservation based on multiple sequence alignments and the images were generated using PyMOL (Figure 1A). Non-conserved residues with high B-factors were selected for mutation (Figure 1B). In addition, ΔΔG was determined using dezyme web server to select mutation sites with negative value. Combining the above two results, 25 mutations D21N, K22P, K22S, P23K, N69D, H71K, K138S, D164S, N166H, H176D, E182D, S75F, E76Y, E76F, E76W, E79Y, E79F, E79W, K160Y, N232F, N232I, N232W, N238F, N238Y, and N238W were selected. Most of the amino acid residues are on the surface of the structure, which maybe effect the thermostability by a new mechanism. The spatial positions of the mutation sites are shown in Figure 1C.

**TABLE 1:** The target residues predicted using HotSpot Wizard 3.0 and dezyme web servers.

Position	WT residue	Mutant residue	B-factor of the residue
21	D	N	37.06
22	K	S	36.55
22	K	P	36.55
23	P	K	32.82
69	N	D	33.05
71	H	K	35.98
138	K	S	32.87
164	D	S	32.73
166	N	H	33.51
176	H	D	34.58
182	E	D	35.73
**Position**	**WT residue**	**Mutant residue**	**∆∆G of the mutant residue**
75	S	F	−1.15
76	E	Y	−1.89
76	E	F	−1.64
76	E	W	−1.55
79	E	Y	−2.11
79	E	F	−1.88
79	E	W	−1.76
160	K	Y	−1.10
232	N	F	−1.59
232	N	I	−1.58
232	N	W	−1.28
238	N	W	−1.42
238	N	F	−1.41
238	N	Y	−1.31

### Protein expression, purification and activity assay of mutants

Except for no expression of K22S, 24 out of 25 mutants were successfully expressed at the correct molecular weight (about 29.86 kDa) (Supplementary Figure S1A). In addition, the mutants N238F, N238Y, N238W and K138S showed insoluble expression (Supplementary Figure S1B). The mutants D21N, N166H, H71K, and H176D had higher expression levels than WT (600 mg/ml).

To evaluate the relative activity of mutants, their catalytic activity was determined as described above. Among the 25 mutants, most of the mutants exhibited activity loss compared to the wild type (97,820 U/mg at optimum testing condition), only four mutants D21N (110%), H71K (110%), E182D (109%), and E79Y (104%) exhibited relative activity above 100% (Supplementary Figure S2).

The specific activity and protein expression levels of WT and some mutants were impressive. A comparison with some reported alginate lyases is necessary. The performance parameters of these alginate lyases are shown in Table 2. The yield (600 mg/L) and specific activity (9.78 × 10^4^ U/mg) of rFlAlyA were higher than known alginate lyases. And the mutants H71K and H176D further improved the yield and thermal stability to some certain.

**TABLE 2:** Catalytic property comparisons of some alginate lyases.

Name	Source	Optimum temperature (°C)	Thermal stability	Specific activity	Yield	Reference
rFlAlyA^H71K^	*E.coli*	50	Retain 25% of activity by incubation at 50°C for 30 min	1.08×10^5^ U/mg^ at 50°C	650 mg/L	This study
rFlAlyA^H176D^	*E.coli*	50	Retain 35% of activity by incubation at 50°C for 30 min	9.0×10^4^ U/mg^ at 50°C	650 mg/L	This study
rFlAlyA^WT^	*E.coli*	50	Retain 20% of activity by incubation at 50°C for 30 min inactivated at 50°C for 30 min	9.78×10^4^ U/mg^ at 50°C	600 mg/L	This study
rFlAlyA	*E.coli*	50		70200 U/mg^ at 30°C	18 mg/L	Inoue et al. (2016b)
Aly5	*E.coli*	40	Retained 80% of activity at 40°C for 2 h	620 U/mg^#^	800 mg/L	Han et al. (2016)
rSAGL	*P. pastoris*	45	Retained 49.0% activity at 50°C for 72 h	226.4 μg/ml	4044 U/mg^※^	Li et al. (2018)
rNitAly	*E.coli*	70	Retain 20% of activity by incubation at 50°C for 16 h	1620 U/mg^	1.2 mg/L	Inoue et al. (2016a)
AlgA	Native	40	Retained 50% activity at 50°C for 0.75 h	8306.7 U/mg^#^	NA	Chen et al. (2018)
AlySY08	Native	40	Retained 75% activity at 40°C for 2 h	1070.2 U/mg^#^	2.1 mg/L	Li et al. (2017)
ALW1	Native	45	Retained 68% of activity at 45°C for 1 h	4.63 U/mg^※^	NA	Zhu et al. (2016)
Alg823	*E.coli*	55	Retained over 75% of the maximum activity at 50°C for 30 min	1.84 U/g^#^	NA	Zeng et al. (2019)

NOTES: Because different reports use different unit definition methods, we distinguished them by using following symbols: ^#^means one unit was defined as the amounts of enzyme that increase the absorbance at 235 nm by 0.1 per min; ^ means one unit was defined as the amounts of enzyme that increase the absorbance at 235 nm by 0.01 per min; ※ means one unit was defined as the amount of enzyme that could release 1 µmol reducing sugar (glucose) per minutes. NA, means not available.

### Thermal stability analysis of mutants

**FIGURE 2:**
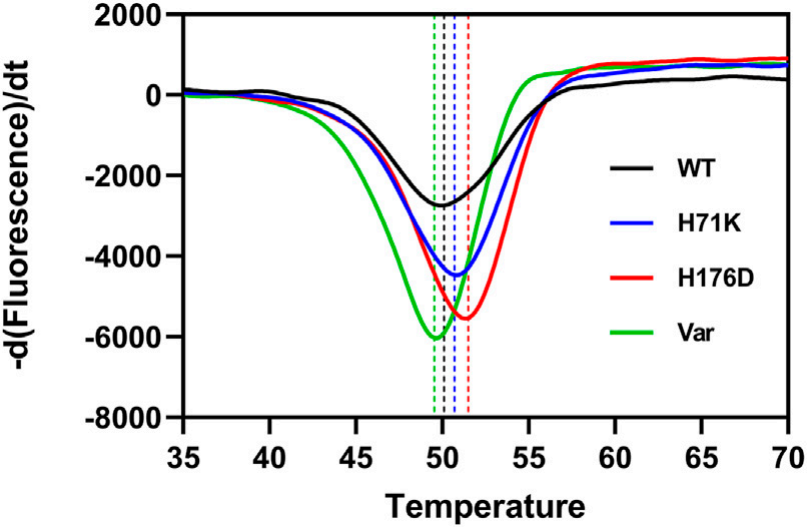
Protein thermal shift analysis of Wild-type and mutants. The positions of the black, blue, red and green dashed lines represent the *T*_m_ values of WT, H71K, H176D, and Var, respectively.

The melting temperature (*T*_m_) was measured to investigate the stability of FlAlyA and its mutants. Compared with WT (50.05°C), two mutants H71K and H176D showed improved *T*_m_ values. The mutants H71K (50.40°C) and H176D (51.31°C) showed 0.35 and 1.26°C improvement of the *T*_m_ value. These shifts in *T*_m_ indicate improved protein stability of the single mutants relative to WT, especially with the H176D mutation. However, the *T*_m_ value of Var (49.69°C) was lower compared to WT (Figure 2).

**FIGURE 3:**
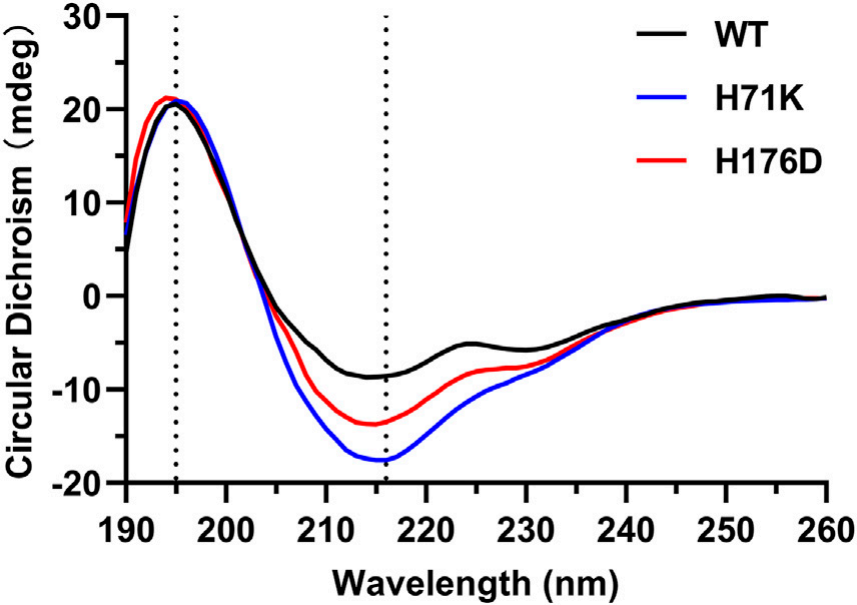
CD characterization of the WT and mutants between 190 and 260 nm. The dotted line indicates the position of the characteristic peaks (195 and 216 nm) of β-sheet.

The impact of H71K and H176D mutations on protein structure was assessed. Comparison of CD spectra showed that the characteristic peaks of β-sheet at 195 and 216 nm existed in WT and mutants, indicates that the secondary structure has not undergone significant changes (Figure 3). The protein structure of the mutant is still intact.

**FIGURE 4:**
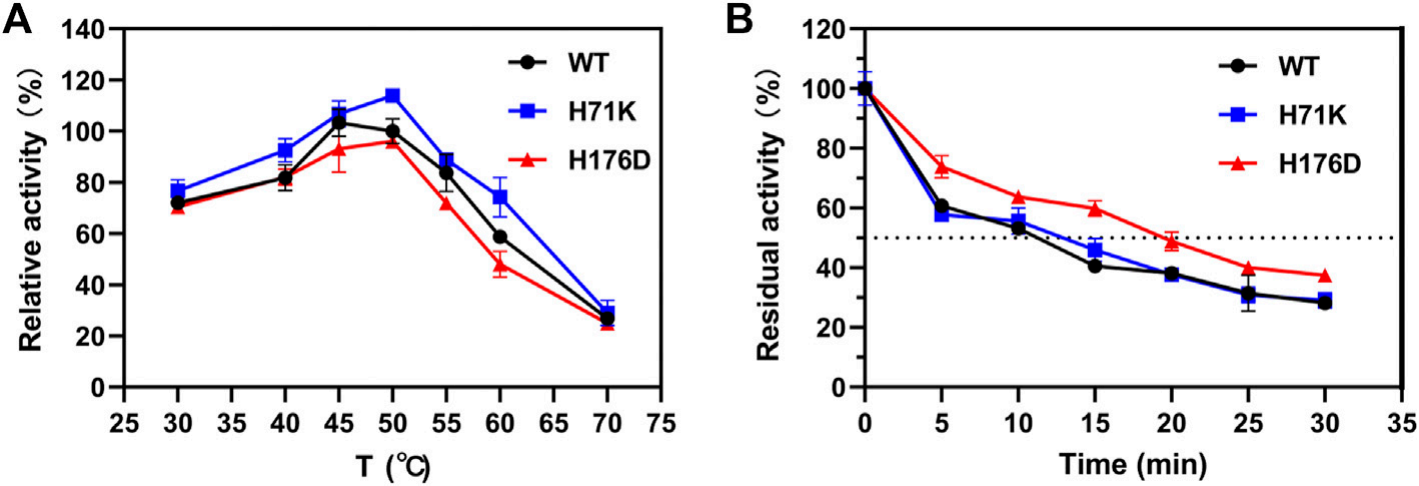
Enzymatic properties of WT and mutants. Effects of temperature on the activity **(A)** and stability **(B)** of WT and mutants. Error bars are standard deviation of three replicates.

The thermostability of mutants was further studied by measuring the optimal temperature (*T*_opt_) and the half-lives (*t*_1/2_). As shown in Figure 4A, *T*_opt_ of both WT and single mutants were 50°C during the temperature range of 30–70°C, the activity of H71K was 1.1 times higher. In addition, the activity of H71K increased to varying degrees at 30–70°C. The thermostability of WT, H71K, and H176D were carried out at 50°C, and their *t*_1/2_ values were 8, 9.73, and 15.58 min (Figure 4B). It is worth noting that the *t*_1/2_ of optimal mutant H176D at 50°C is nearly twice that of WT. This demonstrated that mutants have good performance at high temperature.

### Enzyme kinetic analysis of mutants

The catalytic efficiency of WT and mutants were measured with different concentrations of sodium alginate as the substrate. The maximum velocity (*V*_max_) and Michaelis constant (*K*_m_) of WT and mutants were calculated by nonlinear fitting using Origin. The *V*_max_ of WT, H71K and H176D were 5.62×10^4^, 6.0×10^4^ and 5.48 × 10^4^ U/mg. The apparent *K*_m_ value of WT was 7.27 ± 1.61 mg/ml, while H71K had a lower *K*_m_ value of 6.97 ± 1.36 and H176D had a higher *K*_m_ value of 7.86 ± 1.65 mg/ml, suggesting that H71K possessed a higher substrate affinity than that of WT. Because the protein concentration of WT and mutants was the same, we can infer that the catalytic constant (*k*_cat_) and the catalytic efficiency (*k*_cat_/*K*_m_) of H71K is 1.06-fold higher and 1.11-fold higher than that of WT.

### Sequence-based analysis

The experimental results demonstrated that H71K and H176D had higher thermostability than the WT. As described in the “consensus design” approach, the respective consensus amino acids contributed above average to protein stability compared to non-conserved amino acids. That is, the amino acid that occurs most frequently at a particular position in a homologous structure is considered to contribute more to stability than other residues at the same position (Porebski and Buckle, 2016). Furthermore, in the multiple sequence alignments between FlAlyA and other reported alginate lyases, the residue at position 71 is conserved in most reported alginate lyases (lysine) but different in the sequence of FlAlyA (histidine), and the residue at position 176 usually exists in the form of aspartic acid (Supplementary Figure S3). The above indicates that K71 and D176 are conserved among alginate lyases. It provides potent theoretical support for thermal stability improved of mutants H71K and H176D.

### The free energy of folding (ΔG) analysis

The free energy of folding (ΔG) is an important indicator for judging the thermodynamic stability of a protein (Cao et al., 2019). The difference in free energy of folding between WT and its mutants indicates the mutational effect (Chen et al., 2022). In this study, the ΔΔG of the mutant H71K and H176D were calculated used the DeepDDG web server (http://protein.org.cn/ddg.html). The ΔΔG of mutants H71K and H176D were 0.195 and 0.015 kcal/mol (>0 is stable, <0 is unstable), respectively, indicating that the mutants were more stable than wild type.

### Structural analysis of mutants by crystal structure

**FIGURE 5:**
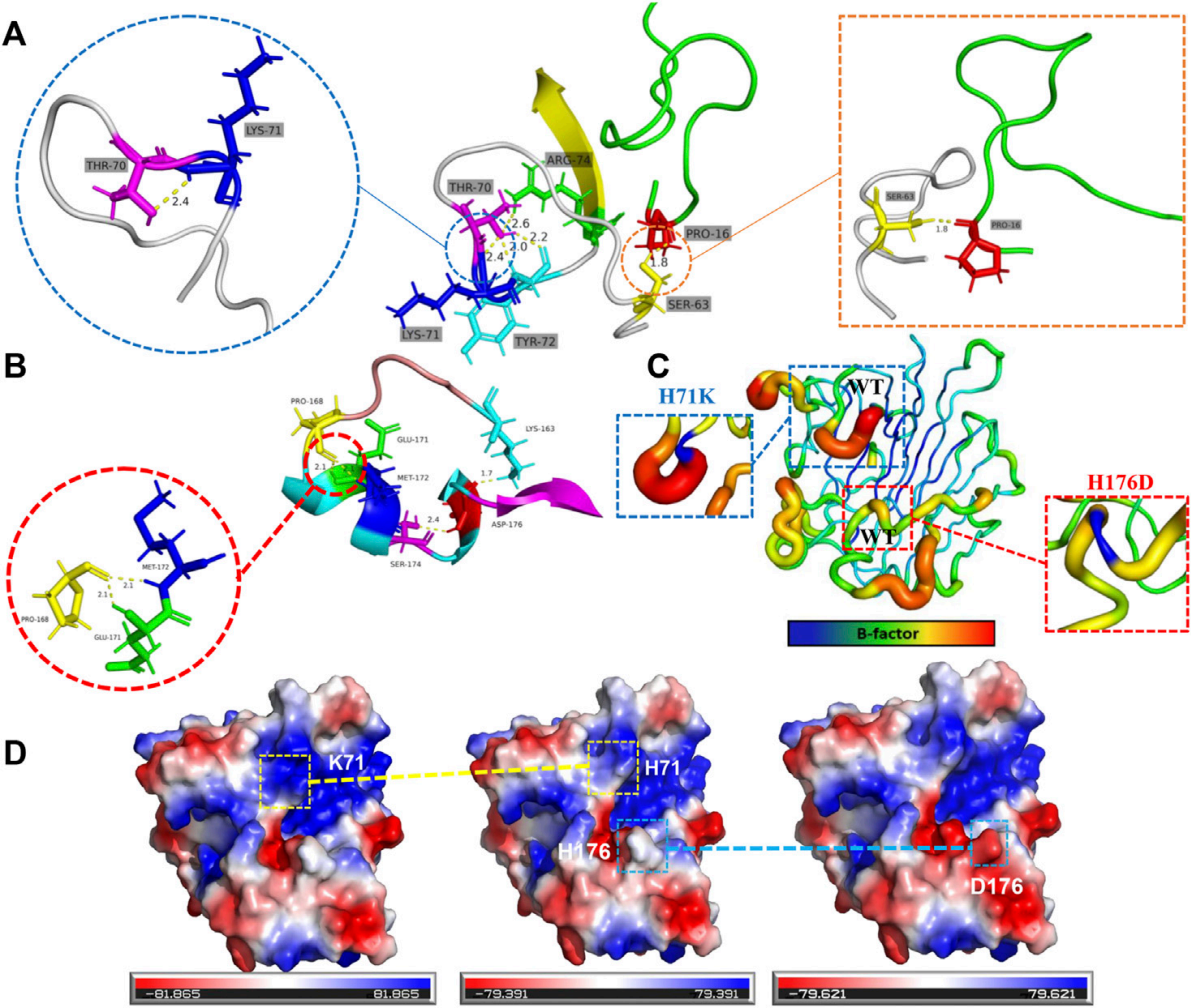
Structural analysis for mutational effects on the thermostability of mutants. Potential hydrogen bonding interactions caused by mutants H71K **(A)** and H176D **(B)** are indicated by yellow dashed lines. **(C)** The effect of mutation on protein B-factor. **(D)** Changes in surface electrostatic charge distribution caused by mutations. The structures shown are H71K, WT, and H176D from left to right.

The structural basis for the improvement of thermostability was further analyzed based on the three-dimensional structures of the mutants that were conducted via PyMol software using structure of 5y33 (PDB ID) as model. As shown in Figure 5A, amino acid residue K71 formed a new hydrogen bond with other residues (T70, Y72, and R74) in the loop F61-S73, which may be beneficial to stabilize the entire loop region. Meanwhile, residue S63 increases the hydrogen bond connection with the adjacent region, which is beneficial to improve the overall rigidity of the protein. Mutant H176D retains the hydrogen bonding of amino acid residue 176 to amino acid residues K163 and S174 while increasing the hydrogen bonding of residue P168 to residues E171 and M172 (Figure 5B). The proline in the loop region usually plays an important role in the stability of the protein (Watanabe and Suzuki, 1998; Huang et al., 2021), and increasing the hydrogen bond connection between the proline 168 and other residues E171 and M172 in the loop region may be of great significance to improve the protein stability. The effect of mutation on protein stability is more intuitively shown in Figure 5C. The protein is stained with PyMOL according to the B-factor value. The B-factor value of the region where the corresponding amino acid residues are located can be reduced by mutation, which is beneficial to stabilize the loop region.

Previous studies have shown that the thermal stability of proteins can be improved by optimizing the distribution of electrostatic charges on the protein surface (Gribenko et al., 2009). Chen et al. constructed a combinational mutant Var3 (G107P/F155Y/D162T/A70P) of *T. caenicola* DAEase, which increased the *T*_m_ value by 12.25°C. Among them, the positive effect of the thermal stability of the mutant D162T may be due to the redistribution of the surface electrostatic charge due to the removal of the carboxyl group (Chen et al., 2022). Therefore, as shown in Figure 5D, the positive effect of mutant H71K on thermal stability may be attributed to the redistribution of surface electrostatic charges caused by the addition of NH_3_^+^. And the mutant H176D redistributed the surface electrostatic charge by the addition of the carboxyl group.

### MD simulation of WT and mutants

**FIGURE 6:**
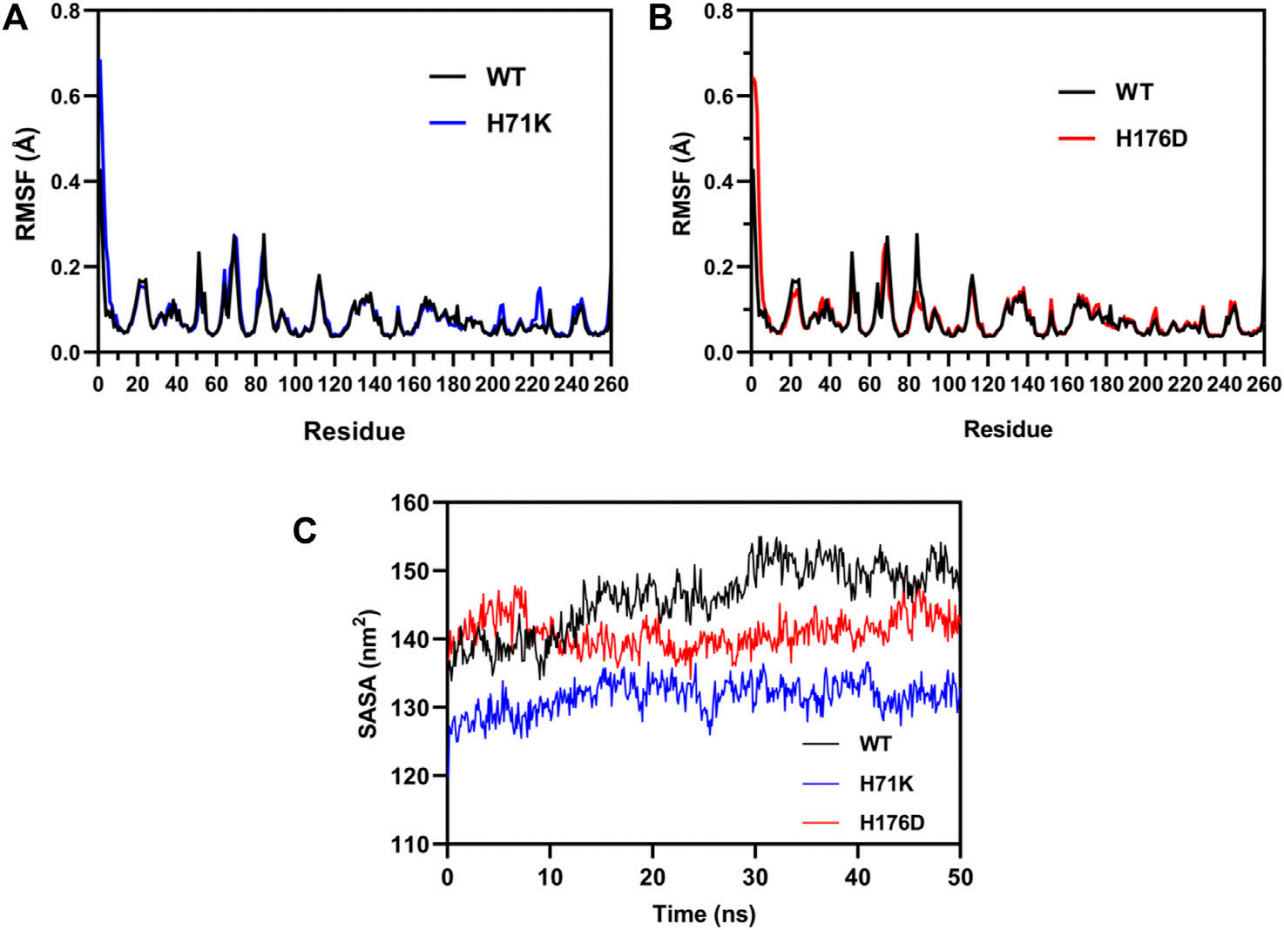
MD simulations for the wild-type enzyme and mutants. RMSF computed from MD simulations for the wild-type enzyme and mutant H71K **(A)** and H176D **(B)** at 300 K for 100 ns. **(C)** SASA of the WT and mutants H71K and H176D changed with simulation time over 50 ns at 323K.

Consequently, MD simulations were performed to investigate the molecular mechanism of the increased thermostability of mutants. The MD simulations were performed at 300 K for 100 ns to calculated the RMSF values for each residue of WT and mutants to investigate changes in structural flexibility. It can be seen from Figures 6A,B that the RMSF value curves of the mutant and the wild type are almost identical, and there is no obvious difference, which may be due to the fact that the wild type enzyme itself is very stable, and a small increase in stability cannot be displayed.

Further, SASA calculated by MD simulations at 323 K for 50 ns. SASA is a parameter used to measure the area of the surface of a protein in contact with the solvent (Durham et al., 2009). High SASA values tend to have low stability. The SASA results showed (Figure 6C) that the amino acids on the protein surface changed over time in an aqueous-dominated solvent environment. The SASA of H71K and H176D remained basically at 130 and 140 nm^2^, respectively, while the SASA of WT remained at 150 nm^2^. The SASA of the mutant is lower than that of the wild type, indicating that the structure of the mutant enzyme is more compact, insensitive to high temperature, not easy to unfold at high temperature, and the solvent molecules did not easily affect the internal structure of the protease, which further improved the overall structural stability of the alginate lyase.

## Conclusion

A high-level expression and thermostable alginate lyase was successfully constructed by computer-aided rational design. Structural analysis and MD simulations showed that the mutants increased the formation of new hydrogen bonds between other residues and decreased the SASA of alginate lyase, thereby improving its performance at high temperature. The two mutants designed in this paper have high expression (650 mg/L), excellent activity (maximum 1.08 × 10^5^ U/mg for H71K and maximum 9.0×10^4^ U/mg for H176D) and good thermal stability (Δ*T*_m_ of H71K and H176D are 0.3 and 1.2°C) and are expected to be used in the industrial production of alginate oligosaccharides. In addition, combining computer design with fusion protein construction (Gao et al., 2015) or DNA recombination library construction (Xu et al., 2021) is becoming a trend for improvement of protein expression levels and thermal stabilities. Therefore, these strategies will be used to further modify the alginate lyase FlAlyA mutants for industrial production in the future.

## Data Availability

The original contributions presented in the study are included in the article/Supplementary Material, further inquiries can be directed to the corresponding authors.
