# Supplementary material for: Improving the thermostability of alginate lyase FlAlyA with high expression by computer-aided rational design for industrial preparation of alginate oligosaccharides
Source: Front Bioeng Biotechnol. 2022 Sep 7;10:1011273. doi: 10.3389/fbioe.2022.1011273 (PMC9490058; doi:10.3389/fbioe.2022.1011273)
Supplement: Supplementary file 1 [file DataSheet1.docx]

Table S1. Mutagenic primers used in this work.

|  |
| --- |

| Number | Mutants | Primer name | Primer sequence (5'-3') |
| --- | --- | --- | --- |
| 1 | D21N | D21N-F | 5'- CTGAGGAGAATCCAaatAAACCAGGTAAGCCATACTCTTTAGG -3' |
|  |  | D21N-R | 5'- attTGGATTCTCCTCAGGCACAGTAACCGTCC -3' |
| 2 | K22S | K22S-F | 5'- TCCAGATagtCCAGGTAAGCCATACTCTTTAGGG -3' |
|  |  | K22S-R | 5'- TACCTGGactATCTGGATTCTCCTCAGGCACA -3' |
| 3 | K22P | K22P-F | 5'- TCCAGATccaCCAGGTAAGCCATACTCTTTAGGG -3' |
|  |  | K22P-R | 5'- TACCTGGtggATCTGGATTCTCCTCAGGCACA -3' |
| 4 | P23K | P23K-F | 5'- aaaGGTAAGCCATACTCTTTAGGGTATCCTGA -3' |
|  |  | P23K-R | 5'- GAGTATGGCTTACCtttTTTATCTGGATTCTCCTCAGGCA -3' |
| 5 | N69D | N69D-F | 5'- CACGGCTgatACGCATTATTCTCGTTCTGAGCT -3' |
|  |  | N69D-R | 5'- CACGGCTgatACGCATTATTCTCGTTCTGAGCT -3' |
| 6 | H71K | H71K-F | 5'- CACGGCTAATACGaagTATTCTCGTTCTGAGCTAAGAGAGACA -3' |
|  |  | H71K-R | 5'- ActtCGTATTAGCCGTGGTCACTCCCGAAGGA -3' |
| 7 | K138S | K138S-F | 5'- TGGTCAAagcGACAACAATGCACCGCCTATTT -3' |
|  |  | K138S-R | 5'- TGTTGTCgctTTGACCAATTAAATCACGTTGTTCA -3' |
| 8 | D164S | D164S-F | 5'- AAAagtTTGAACGCGCCTTATAAAGAAATGCT -3' |
|  |  | D164S-R | 5'- GGCGCGTTCAAactTTTAAGTACTTTTGTTTTCACACGAATT -3' |
| 9 | N166H | N166H-F | 5'- AGATTTGcacGCGCCTTATAAAGAAATGCTTTC -3' |
|  |  | N166H-R | 5'- AAGGCGCgtgCAAATCTTTAAGTACTTTTGTTTTCACACG -3' |
| 10 | H176D | H176D-F | 5'- TTCAGAAgatGCTTGGGGTGATGATGAAGGTC -3' |
|  |  | H176D-R | 5'- CCCAAGCatcTTCTGAAAGCATTTCTTTATAAGGCG -3' |
| 11 | E182D | E182D-F | 5'- GTGATGATgatGGTCGAAATTTTAAAGAGAAAATCG -3' |
|  |  | E182D-R | 5'- TCGACCatcATCATCACCCCAAGCATGTTCTG -3' |
| 12 | S75F | S75F-F | 5'- TCTCGTtttGAGCTAAGAGAGACAATGGAAACTGG -3' |
|  |  | S75F-R | 5'- CTTAGCTCaaaACGAGAATAATGCGTATTAGCCG -3' |
| 13 | E76Y | E76Y-F | 5'- TCGTTCTtacCTAAGAGAGACAATGGAAACTGGTAGC -3' |
|  |  | E76Y-R | 5'- CTCTTAGgtaAGAACGAGAATAATGCGTATTAGCC -3' |
| 14 | E76F | E76F-F | 5'- TCGTTCTttcCTAAGAGAGACAATGGAAACTGGTAGC -3' |
|  |  | E76F-R | 5'- CTCTTAGgaaAGAACGAGAATAATGCGTATTAGCC -3' |
| 15 | E76W | E76W-F | 5'- TCGTTCTtggCTAAGAGAGACAATGGAAACTGGTAGC -3' |
|  |  | E76W-R | 5'- CTCTTAGccaAGAACGAGAATAATGCGTATTAGCC -3' |
| 16 | E79Y | E79Y-F | 5'- GCTAAGAtacACAATGGAAACTGGTAGCAATAAGG -3' |
|  |  | E79Y-R | 5'- CCATTGTgtaTCTTAGCTCAGAACGAGAATAATGCG -3' |
| 17 | E79F | E79F-F | 5'- GCTAAGAttcACAATGGAAACTGGTAGCAATAAGG -3' |
|  |  | E79F-R | 5'- CCATTGTgaaTCTTAGCTCAGAACGAGAATAATGCG -3' |
| 18 | E79W | E79W-F | 5'- GCTAAGAtggACAATGGAAACTGGTAGCAATAAGG -3' |
|  |  | E79W-R | 5'- CCATTGTccaTCTTAGCTCAGAACGAGAATAATGCG -3' |
| 19 | K160Y | K160Y-F | 5'- CGTGTGAAAACAtacGTACTTAAAGATTTGAACGCGCC -3' |
|  |  | K160Y-R | 5'- ACgtaTGTTTTCACACGAATTTTTCCTTTATC -3' |
| 20 | N232F | N232F-F | 5'- GGGGGATATTCGAAtttTATTTCAAAGCAGGGAATTATTTTCA -3' |
|  |  | N232F-R | 5'- aaaTTCGAATATCCCCCATTTTTTCATGTGAA -3' |
| 21 | N232I | N232I-F | 5'- GGGGGATATTCGAAatcTATTTCAAAGCAGGGAATTATTTTCA -3' |
|  |  | N232I-R | 5'- gatTTCGAATATCCCCCATTTTTTCATGTGAA -3' |
| 22 | N232W | N232W-F | 5'- GGGGATATTCGAAtggTATTTCAAAGCAGGGAATTATTTTCA -3' |
|  |  | N232W-R | 5'- AccaTTCGAATATCCCCCATTTTTTCATGTGA -3' |
| 23 | N238W | N238W-F | 5'- AGCAGGGtggTATTTTCAATCTAAAACACCAGGGAC -3' |
|  |  | N238W-R | 5'- GAAAATAccaCCCTGCTTTGAAATAATTTTCGA -3' |
| 24 | N238F | N238F-F | 5'- AGCAGGGtttTATTTTCAATCTAAAACACCAGGGAC -3' |
|  |  | N238F-R | 5'- GAAAATAaaaCCCTGCTTTGAAATAATTTTCGA -3' |
| 25 | N238Y | N238Y-F | 5'- AGCAGGGtatTATTTTCAATCTAAAACACCAGGGAC -3' |
|  |  | N238Y-R | 5'- GAAAATAataCCCTGCTTTGAAATAATTTTCGA -3' |
| 26 | Var | Var-F | \| 5'- TTCAGAAgatGCTTGGGGTGATGATGAAGGTC -3' \| \| --- \| |
|  |  | Var-R | \| 5'- CCCAAGCatcTTCTGAAAGCATTTCTTTATAAGGCG -3' \| \| --- \| |

Note: The mutated amino acids were in lower case.


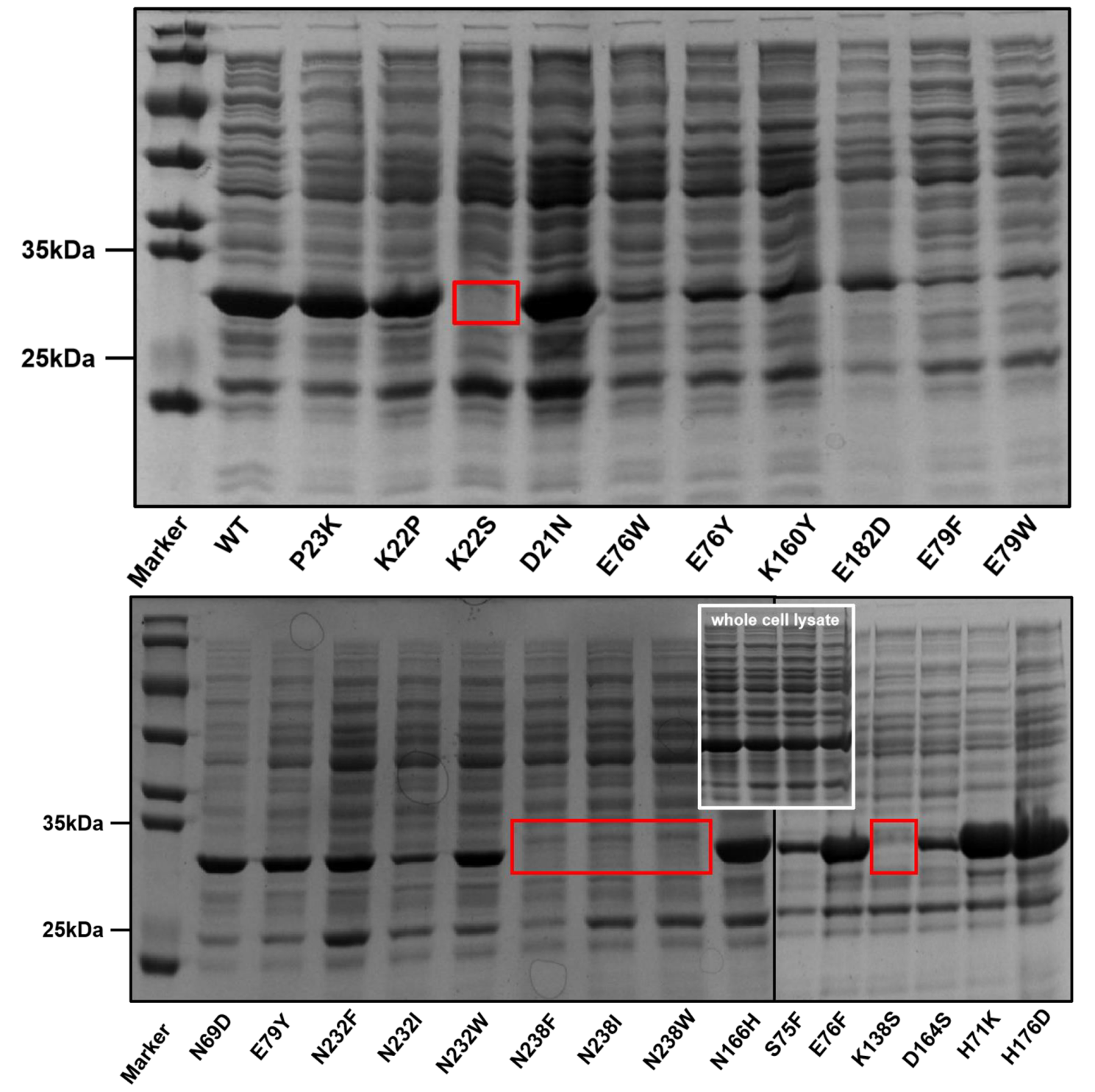
Figure S1. SDS-PAGE analysis of the WT and mutants. (A) SDS-PAGE analysis of whole cell lysate of BL21(DE3); (B) SDS-PAGE analysis of supernatant of whole cell lysate with recombinant vectors. M: protein marker. Whole cell lysates of N232F, N232I, N232W, and K138S are represented in white boxes from left to right.


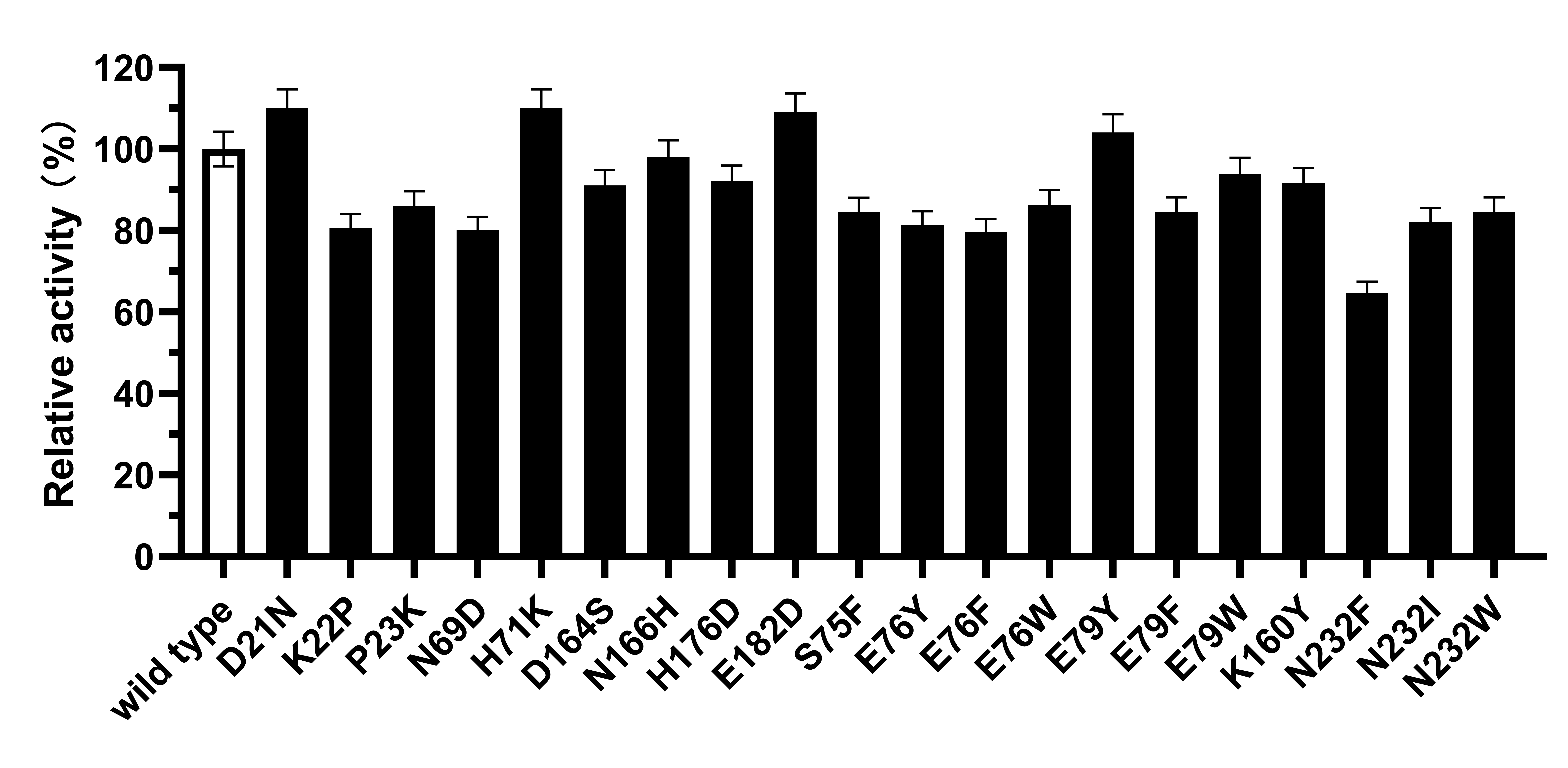


Figure S2. The relative activities of the mutants. All the mutants were incubated at 30℃, the relative activity of wild type was identified as 100%


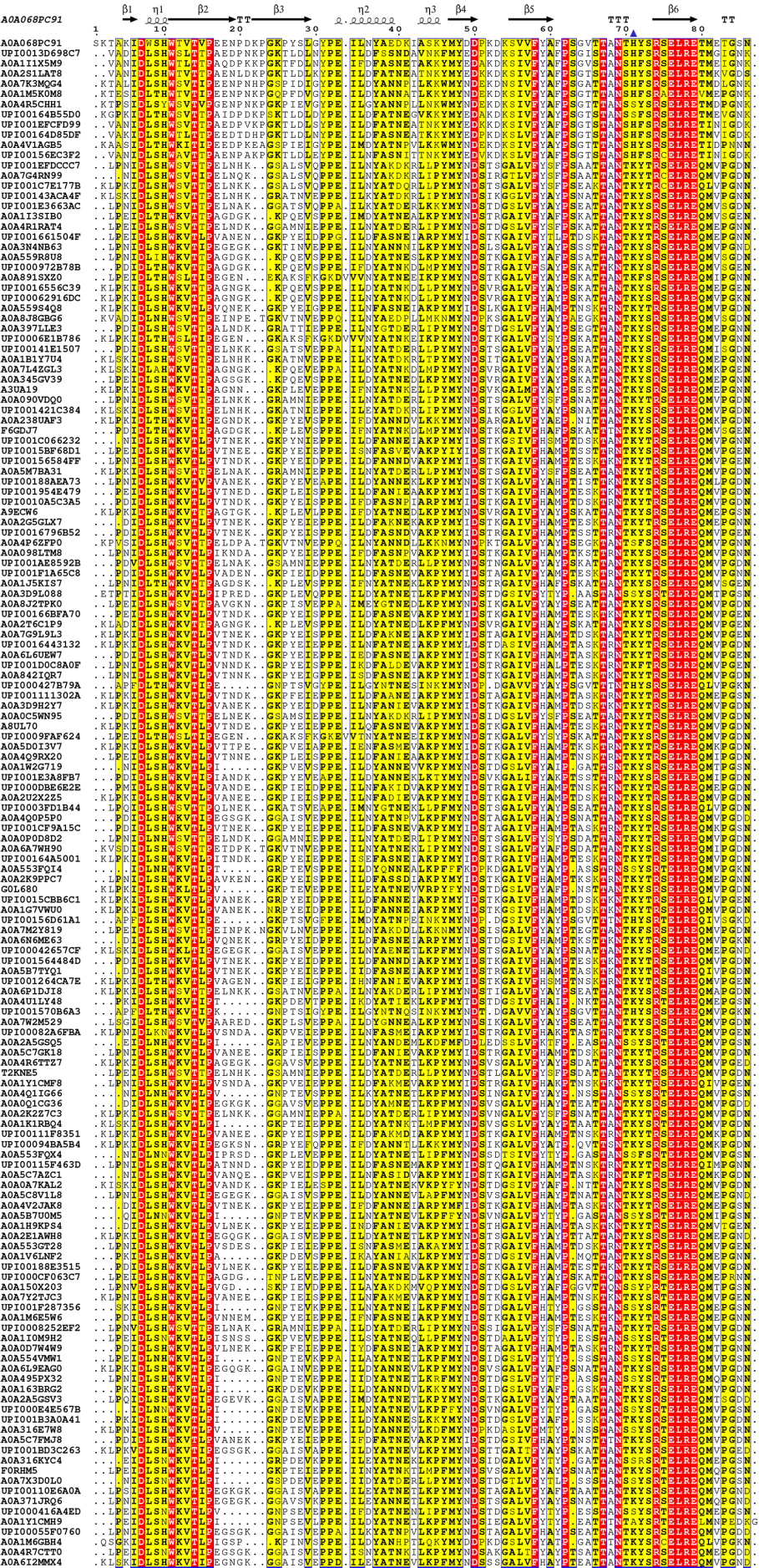

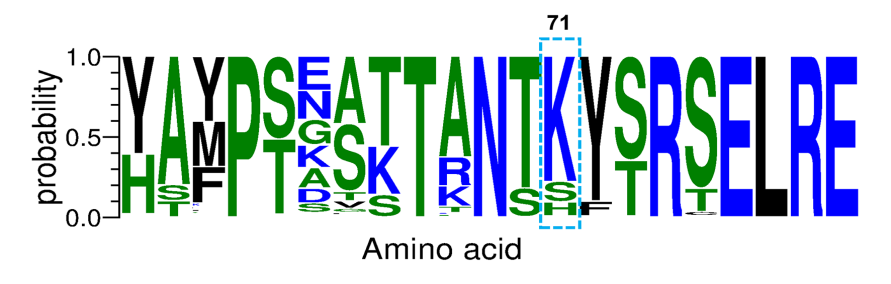


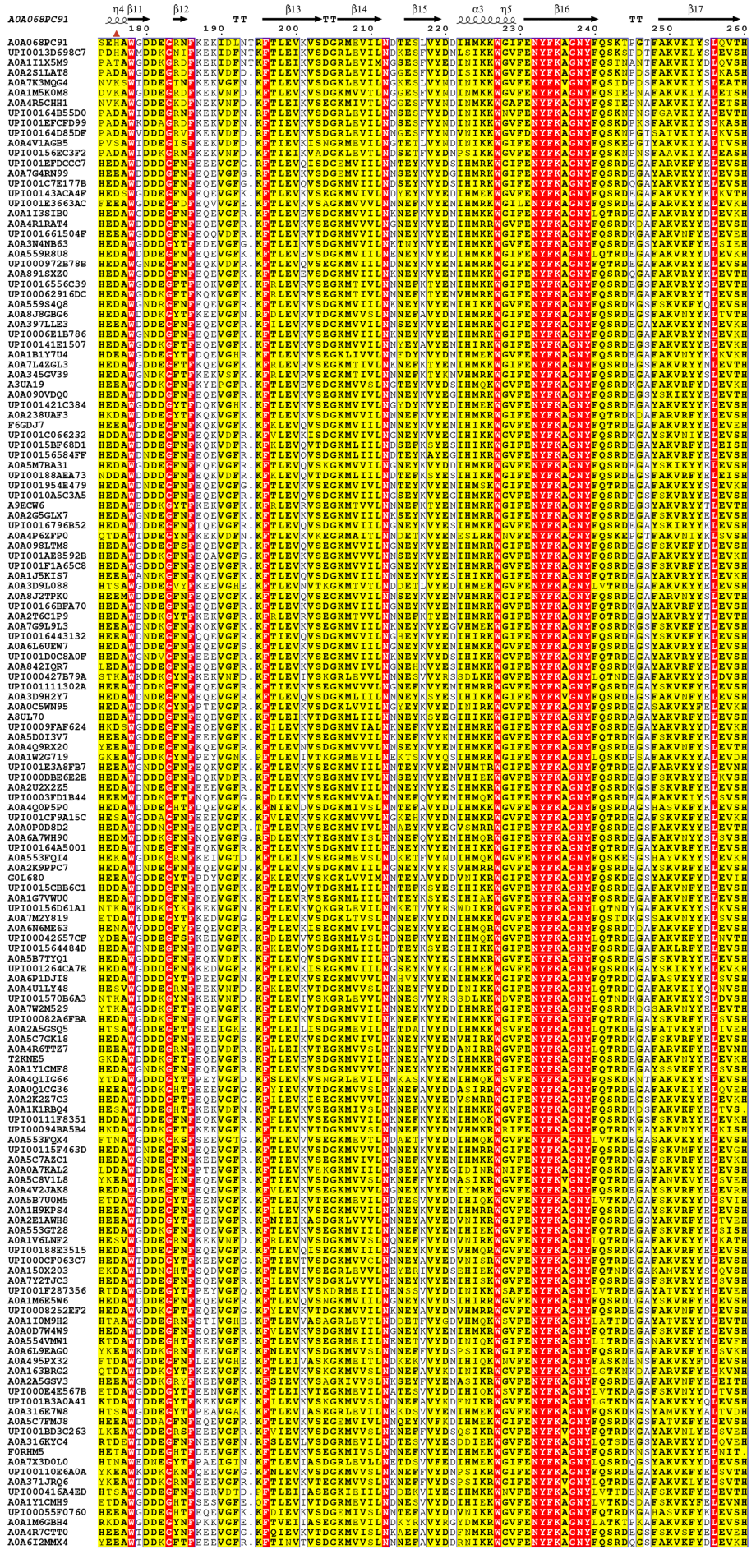

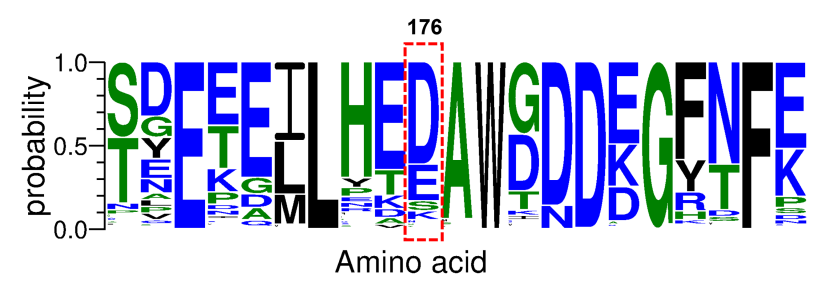


Figure S3. Multiple sequence alignment of FlAlyA with other alginate lyases. The sequences were obtained from UniProt. UniProt accession number of FlAlyA is A0A068PC91. The secondary structure of FlAlyA is shown above the alignment. A “·” is a gap. The blue solid triangle and the red solid triangle represent residue 71 and residue 176, respectively. The alignment was performed using CLUSTALW, and the image was prepared using ESpript 3 and WebLogo 3.
